# The Relationship between Self-esteem and Quality of Life of Patients with Idiopathic Thrombocytopenic Purpura at Isfahan's Sayed Al-Shohada Hospital, Iran, in 2013

**Published:** 2016-04-01

**Authors:** Zeinab Hemati, Davood Kiani

**Affiliations:** 1 PhD Candidate of Nursing, Nursing and Midwifery Care Research Center, Faculty of Nursing and Midwifery, Isfahan University of Medical Sciences, Isfahan, Iran; 2 BSc of Nursing, Shahrekord University of Medical Sciences, Shahrekord, Iran

**Keywords:** Self-esteem, Quality of life (QOL), Idiopathic thrombocytopenic purpura (ITP), Nurse, Iran

## Abstract

**Background:** Idiopathic thrombocytopenic purpura (ITP) is a chronic disease which is accompanied with hopelessness and loss of the sense of well-being due to its symptoms and treatment. It also affects patients' sense of social and spiritual well-being. This disorder decreases patients' self-esteem and their quality of life by changing their mental image and self-confidence. This study was performed to find the relationship between self-esteem and quality of life of patients with ITP.

**Subjects and Methods:** This was a descriptive-analytical study on 64 patients with ITP who referred to Isfahan's Sayed Al-Shohada Hospital, Iran. In this study, patients with ITP were selected randomly using a random number chart. The data collection tools consisted of the World Health Organization Quality of Life (WHOQOL)-BREF and Coopersmith Self-esteem Inventory (CSEI). Data were analyzed using SPSS and chi-square and Mann-Whitney tests and the Pearson and Spearman’s rank correlation coefficients.

**Results:** In total, 64 patients completed the questionnaires. Results showed that **32**% of subjects were over 36 years of age and 59% were women. In addition, 29.7% of ITP patients had low self-esteem and quality of life. Chi-square test showed a significant relationship between self-esteem and quality of life of patients with ITP.

**Conclusions:** The results of the present study showed that considerable attention must be paid to self-esteem, as one of the most important factors influencing the promotion of quality of life. Therefore, it is suggested that patient’s self-esteem be improved by the implementation of educational and psychological programs in order to decrease the consequences of poor quality of life.

## Introduction

 Idiopathic thrombocytopenic purpura (ITP) is a chronic autoimmune disorder accompanied with bleeding. Decreased number of platelets due to their increased destruction is the main manifestation of the disease.^1^ This disease is the most prevalent autoimmune disorder involving blood cells.^2^ The prevalence of this disease is 2 to 5 children in every 100 and 2 to 6 adults in every 100 and 61% in women and 38% in men.^3^ Worldwide, 4 to 7 million people have the chronic form of this disease. The highest prevalence rates of this disease are observed in Australia^4^ and Canada.^5^ In contrast to its acute form, which is benign and self-limited, the chronic form is accompanied by permanent thrombocytopenia. Only in less than one third of chronic cases of this disease recovery is observed without any treatment after some years.^6^

The most significant complication caused by this disease is the decrease in the number and function of platelets which causes bleeding.^7^ Bleeding is mostly seen in the skin and mucosa.^8^ Claire et al. found in their study that the most important complications of this disease include petechia, anemia and extensive bleeding. These patients should be monitored regularly to measure the level of platelets.^8^ As a result of these symptoms, symptoms such as weakness, fatigue, decreased energy level and psychological changes are observed in these patients.^9^

The psychological changes are mainly due to the reduction of personal communications and limitations in daily activity because of fatigue. The patient’s sense of shame and humiliation because of the observable signs of this disease causes a decrease in the social interactions of the patient.^10^ This decrease in interactions can affect patients’ self-esteem, level of activity, happiness and quality of life (QOL).^11^

Self-esteem is defined as an individual’s sense of self-worth and acceptance. An individual with high self-esteem evaluates him/herself positively and communicates efficiently with other people. Self-esteem is composed of two related parts; the first is the feeling of self-assurance in handling life challenges and believing in one’s ability and the second part includes believing to be deserving of success and happiness and having self-respect.^12^

Patients with low self-esteem usually focus on their negative points and spend less time thinking of their positive aspects. However, it is necessary to identify both strengths and weaknesses. Since, self-esteem has a unique role in the QOL of chronic patients and controlling disease complications, studying it is very crucial. To our knowledge, no similar study has been performed on the self-esteem of chronic patients.^13^ The study by Mc Millan et al. showed that bleeding, side effects of corticosteroid medications, changes in appearance and fear of bleeding and infection after splenectomy cause a decrease in the psychological dimension of the quality of life of these patients.^14^ The results of the study by von et al. showed that symptoms such as bleeding, weakness, fatigue, pain and reduction of energy level are factors which decrease the physical dimension of the quality of life of patients.^9^

Therefore, the present study was conducted to determine the relationship between self-esteem and quality of life of ITP patients. We hope that officials utilize the results of this project in educational, research, and treatment fields in order to improve these patients treatment process, promote their self-esteem and thus, increase their quality of life and health.

## SUBJECTS AND METHODS

 This is a descriptive-analytical study in which 64 ITP patients participated. The approval of the Vice Chancellor for Research and the Ethics Committee of the Isfahan University of Medical Sciences, Iran, and a written permission from the officials were obtained.(Ethical code: 288250) Then, subject who had the inclusion criteria of the study and referred to the Sayed Al-Shohada Hospital were randomly selected, according to random numbers chart. The inclusion criteria consisted of being 20 to 70 years old, living in Isfahan, being diagnosed conclusively as having ITP based on medical and laboratory findings (6 months had passed since their diagnosis), lacking of mental and cognitive problems and not experiencing a stressful event such as losing a relative during the previous month.

It is supposed that any subjects who participated in this project had a conclusive diagnosis and 6 months had passed since the diagnosis (chronic disease is defined as a disease which affects daily life and at least, 3 months have passed since the diagnosis). Exclusion criteria comprised of the lack of willingness to take part in the study. After receiving written consent from the patients and describing the study goals to them, the researcher began completing the questionnaires. Quality of life, self-esteem and ITP were the main variables and sex, age, educational level, illness duration, and hospitalization frequencies were contextual variables. The data collection instrument was the World Health Organization Quality of Life (WHOQOL)-BREF questionnaire which is the summarized form of the comprehensive 100-question quality of life measurement. It consists of 26 questions, scored on a 5-point Likert scale, which monitor different aspects of an individual's quality of life. In this questionnaire, two questions were related to the patients’ general feelings about their quality of life. The remaining questions were related to the patients’ feelings and behaviors during the previous 2 weeks in the physical dimension (physical activities, drug dependency and supportive medicines, mobility, pain and feeling of discomfort, sleep and rest and the ability to perform activities), psychological dimension (feeling toward body posture and appearance, positive and negative feelings, learning, thoughts, memory and concentration, self-confidence and personality traits), social dimension (personal relationships, social support), and environmental dimension (financial sources, freedom and physical security, accessibility to social and health care's, house condition, available chances, accessibility to new data and various skills, opportunity to take part in social activities and physical environment such as pollution, noise, traffic, and transportation). One question was related to the sexual dimension. Every question has a score range from 0 to 4; 0 represented the worst and 4 represented the best conditions of quality of life. Achieved scores were converted into 100 in every dimension. The least and most scores, in any dimension, were 0 and 100, respectively. This questionnaire has been prepared and compiled through the cooperation of 15 international departments in order to be used in different cultures.^15^ In addition; its validity has been approved in the study performed by Yousefy et al. in Iran.^16^

The Coopersmith Self-esteem Inventory (CSEI) was applied to study patients' self-esteem level. This inventory is one of the most famous instruments in measuring self-esteem and has been used frequently. This scale consists of 58 questions 26 of which were related to general, social, familial, and professional issues. In addition, 8 questions were specified for fake replies which, in fact, are considered as a sign of a defensive reaction toward the questionnaire.

Therefore, the lowest and highest scores are 0 and 50, respectively. The closer the score is to 50, the higher the level of self-esteem and vice versa. The test-retest reliability coefficients of the CSEI have been respectively reported as 0.91 and 0.73. The validity and constancy of this instrument has been found to be 92% in previous research which reflects the constancy, reproducibility, and accuracy of this instrument.^17^ Delaram et al. calculated the validity of the CSEI by test-retest method at 90% and 93%, respectively.^18^ Data were analyzed by descriptive-analytical statistics and using SPSS for Windows (version 15; SPSS Inc., Chicago, IL, USA). Chi-square test was used to compare the relationship between self-esteem and quality of life in patients with ITP.

## Results

 The results showed that 32% of patients were 36 and older, 59% were female and 26.6% had primary education. The majority of ITP patients (29.7%) had low self-esteem and quality of life. Chi-square test showed a significant relationship between self-esteem and quality of life of ITP patients (p = 0.03) (Table 1). Pearson’s correlation coefficient showed no significant relationship between mean total score of self-esteem of patients and their demographic variables (age, hospitalization frequency). However, it showed a significant relationship between self-esteem and duration of illness (r = 0.26, p < 0.03) and education level (r = 0.36, p < 0.003).

## Discussion

 Self-esteem and self-respect are regarded as a crucial value and are necessary factors in the flourishing of talent and creativity. In other words, cognitive process, feelings, motivation, decision making and selection are all affected by the feeling of self-respect. Low self-esteem, especially in chronic diseases, has a negative impact on patients' interpersonal relations, thoughts, feelings, and functioning.^19^ Most subjects, in present study, were older than 36 and female. The results of the study by Zhou et al. showed that 77% of the studied subjects were female and their average age was 45 years.^20^

Previous researches show that people with chronic diseases are of older age.^21^^,^^22^ This is in agreement with the results of the present study. Most of the patients had primary education level, which can be the result of the disease complications and their negative impacts on the patients’ lifestyle.

**Table 1:** Frequency distribution and relationship between self-esteem and quality of life in patients with ITP

**Quality of life**	**Very inappropriate** **(0-25)**	**Inappropriate** **(26-50)**	**Appropriate** **(51-75)**	**Very Appropriate** **(76-100)**	**Total**

**Self-esteem**	No (%)	No (%)	No (%)	No (%)	No (%)
Low	3 (4.7)	19 (29.7)	14 (21.9)	1 (1.6)	37 (57.8)
High	3 (4.7)	4 (6.3)	17 (26.6)	3 (4.7)	27 (42.2)
Total	6 (9.4)	23 (35.9)	31 (48.4)	4 (6.3)	64 (100)

These complications reduce the patients’ opportunities of advancement and development.^20^ The results show that the duration of illness is one of the important variables which have a direct effect on patients' self-esteem; in addition to changes in QOL, the long-term treatment of ITP affects their self-esteem.

Patients suffering from chronic diseases, due to the feeling of loss of self-control, are prone to low self-esteem, which in turn will result in reduction of quality of life and increase in prevalence of depression and other physical diseases.^23^

The results of the present study showed that there was a significant relationship between self-esteem and quality of life of ITP patients. The majority of ITP patients have low self-esteem and quality of life. Platelet level reduction causes low energy and activity levels and thus, results in the reduction of patients functioning level. Claire et al. showed that Cushing disease symptoms and changes in the patients' appearance after long-term use of corticosteroid drugs reduce the mental dimension of quality of life of patients.^8^ This in turn, leads to a decrease in patients' self-esteem.

The most important causes of self-esteem reduction consist of quality of life reduction after hospitalization, functioning impairment, prolonged use of medicines with adverse side effects, changes in appearance, and, subsequently, changes in the patients' psychological state. Chia–Huei showed that daily events and chronic diseases affect self-esteem level. They also found that sever stress related to chronic diseases can decrease self-esteem considerably.^24^

Therefore, psychologists consider accepted social roles and patterns as one of the main parts of self-esteem and believe that inability to or limitation in playing these roles and patterns can decrease self-esteem level.^25^ Therefore, family support and intimate relations with other people can induce an appropriate level of self-esteem.

The results of the present study illustrated that there was a significant relation between self-esteem and hospitalization frequencies and duration. The results of the study by Sharifi Neyestanak et al. were in agreement with this finding.^25^ Fear of patent bleeding and long term hospitalization leads to diminished self-esteem and quality of life of patients.^14^

Patients' ages and morbidity duration are factors effective in the understanding of psychological variables.^26^ Disorder in any dimensions of mental health influences other dimensions. Therefore, older patients with longer illness duration have lower levels of mental health.^27^ The impact of ITP, its treatment and complications and long-term hospitalization on self-esteem is unquestionable. Moreover, patients' self-esteem influences their functioning and different dimensions of quality of life. Thus, the treatment team must improve the QOL of patients by identifying factors effective on improvement of self-esteem and reinforcing feelings of self-respect. The limitation of the present study consisted of the limited number of studied subjects. It is suggested that future projects be performed on more subjects and their results be compared with the results of the present study.

## CONCLUSION

 It is recommended that the self-esteem level of ITP patients be raised by the implementation of psychiatric interventions.

Thus, the complications resulting from low self-esteem and its effect on quality of life of patients, and the psychological problems and desocialization of the patient can be prevented.
